# GABA_B_ receptor-mediated activation of astrocytes by gamma-hydroxybutyric acid

**DOI:** 10.1098/rstb.2013.0607

**Published:** 2014-10-19

**Authors:** Timothy Gould, Lixin Chen, Zsuzsa Emri, Tiina Pirttimaki, Adam C. Errington, Vincenzo Crunelli, H. Rheinallt Parri

**Affiliations:** 1 Neuroscience Division, School of Biosciences, Cardiff University, Museum Avenue, Cardiff CF10 3AX, UK; 2 School of Life and Health Sciences, Aston University, Birmingham B4 7ET, UK

**Keywords:** ventral tegmental area, thalamus, baclofen, reward, absence seizures

## Abstract

The gamma-aminobutyric acid (GABA) metabolite gamma-hydroxybutyric acid (GHB) shows a variety of behavioural effects when administered to animals and humans, including reward/addiction properties and absence seizures. At the cellular level, these actions of GHB are mediated by activation of neuronal GABA_B_ receptors (GABA_B_Rs) where it acts as a weak agonist. Because astrocytes respond to endogenous and exogenously applied GABA by activation of both GABA_A_ and GABA_B_Rs, here we investigated the action of GHB on astrocytes on the ventral tegmental area (VTA) and the ventrobasal (VB) thalamic nucleus, two brain areas involved in the reward and proepileptic action of GHB, respectively, and compared it with that of the potent GABA_B_R agonist baclofen. We found that GHB and baclofen elicited dose-dependent (ED_50_: 1.6 mM and 1.3 µM, respectively) transient increases in intracellular Ca^2+^ in VTA and VB astrocytes of young mice and rats, which were accounted for by activation of their GABA_B_Rs and mediated by Ca^2+^ release from intracellular store release. In contrast, prolonged GHB and baclofen exposure caused a reduction in spontaneous astrocyte activity and glutamate release from VTA astrocytes. These findings have key (patho)physiological implications for our understanding of the addictive and proepileptic actions of GHB.

## 1. Introduction

Gamma-hydroxybutyric acid (GHB) is an endogenous central nervous system (CNS) substance that results from the metabolism of the neurotransmitter gamma-aminobutyric acid (GABA) [1–3], but can also act as a source of neuronal GABA [4]. In humans, exogenously administered GHB elicits a variety of behavioural responses that at progressively increasing doses include sedation, memory loss, euphoria, behavioural disinhibition, sleep and coma [2,5–8]. GHB has also found some limited clinical use in the treatment of alcohol and opiate withdrawal [9–12] as well as of narcolepsy and cataplexy [13–15]. In the early 1970s, GHB emerged as a recreational drug and still remains one of the most commonly used ‘club drugs’ [16–19], with GHB overdoses accounting for a substantial proportion of the hospital emergencies that are linked to recreational nightlife settings [20].

A neuropharmacological profile similar to that observed in humans characterizes the behaviour of animals injected with GHB. The most extensively studied behavioural actions of GHB are its ability to induce self-administration and absence seizures [3]; indeed, the systemic administration of low doses of GHB has become the most widely used pharmacological model of these non-convulsive seizures [21]. Thus, oral GHB induces self-administration and conditioned place preference in mice and rats [22–25]. In addition, GHB administration leads to a moderate stimulation of the dopaminergic reward system [26], and *in vitro* low concentration of GHB preferentially inhibits the GABAergic neurons of the ventral tegmental area (VTA), one of the key brain-reward areas [25,27]. Indeed, GHB induces conditioned place preference when injected directly in the VTA [28], but not in the nucleus accumbens, another important brain-reward area. As far as absence seizures are concerned, the ability of GHB to elicit absence seizures is not restricted to its systemic administration but importantly it also occurs when it is injected directly into the ventrobasal (VB) nucleus of the thalamus [29], one of the key areas for the generation of these non-convulsive seizures [30].

In both mice and rats, all behavioural and cellular actions of GHB, including its reward- and seizure-eliciting properties, can be explained by it acting as a weak agonist at GABA_B_ receptors (GABA_B_Rs) [31,32], though the existence of a putative GHB receptor site has been suggested [33] (but see [3]). Thus, the pro-absence action of GHB is mimicked by the selective GABA_B_R agonist baclofen and is fully antagonized by different selective GABA_B_R antagonists [3], and in some studies by NCS382, an antagonist of the putative GHB receptor site [34]. Interestingly, micromolar GHB concentrations have recently been suggested to activate recombinant α4βδ GABA_A_Rs [35], though the physiological significance of this finding remains obscure as these results could not be reproduced in different brain areas, including thalamus, cerebellum and hippocampus [36] that contain this combination of GABA_A_R subunits.

It is now well-established that astrocytes respond to different neurotransmitters, including GABA [37,38] and signal back to neurons via the release of gliotransmitters [39,40], thus modulating neuronal excitability and different forms of synaptic plasticity [41]. Thus, because astrocytes respond to GABA_B_R activation [42], and the VTA and VB thalamus contribute to the behavioural actions of GHB [3], we investigated the effect of GHB on this glial cell type in brain slices and compared it with the selective GABA_B_R agonist baclofen. We report that GHB and baclofen elicit a dose-dependent increase of intracellular Ca^2+^ ([Ca^2+^]_i_) in VTA and VB astrocytes which is fully accounted for by activation of their GABA_B_Rs and is mediated by Ca^2+^ release from intracellular stores. In contrast to many other studies which found that transient as well as sustained agonist-induced astrocytic [Ca^2+^]_i_ elevations result in increased gliotransmitter release, prolonged GHB and baclofen exposure causes a reduction in spontaneous glutamate release from VTA astrocytes.

## 2. Materials and methods

All experiments were performed in accordance with the Animals (Scientific Procedures) Act 1986, UK, and with approval of local animal welfare and ethical review bodies.

### (a) Slice preparation and maintaining solutions

Male and female Wistar rats (8–12 days old), as well as GABA_B_ KO mice and their wild-type littermates (kindly provided by B. Bettler, Basel, Switzerland) [43] were deeply anaesthetized with isoflurane, and the brain quickly removed. Slices of VTA or VB thalamus were prepared as described previously [44,45]. Briefly, the brain was glued with cyanoacrylate adhesive to a metal block and submerged in the bath of a Leica or Microm MV tissue slicer. The bathing solution was of composition (in mM): NaCl 120, NaHCO_3_ 16, KCl 1, KH_2_PO_4_ 1.25, MgSO_4_ 5, CaCl_2_ 1, 10 glucose, and was maintained at 5°C. Slices (350 µm) were cut in the horizontal plane, and then stored in a 95% O_2_, 5% CO_2_-bubbled solution of identical composition at room temperature. Following a 1 h recovery period, experiments were performed in a solution of composition (in mM): NaCl 120, NaHCO_3_ 16 or 25, KCl 2, KH_2_PO_4_ 1.25, MgSO_4_ 1, CaCl_2_ 2, 10 glucose, at room temperature (20–24°C), unless otherwise stated. Slices were loaded with Fluo-4 AM (Invitrogen, Paisley, UK) by incubating for 40–60 min at 28°C with 5 μM of the indicator dye and 0.01% pluronic acid. TTX (1 µm) was present in the perfusate of all experiments. Agonists (GHB and baclofen) were bath-applied for 2 min with or without GABA_B_R antagonists (unless stated otherwise). Chemicals were purchased from Sigma (St Louis, MO), except CGP65426, MTEP, CNQX, CPCCOEt and baclofen (Tocris, Bristol, UK), D-APV and TTX (Abcam, Cambridge, UK), and GHB (that was kindly donated by Unavera ChemLab GmbH, Mittenwald, Germany).

### (b) Fluorescence imaging

The slices were placed in a recording chamber, whereas the patch-electrode headstage micromanipulators were mounted on a movable platform (MP MTP-01, Scientifica, UK). Fluorescence was measured using a Noran Odyssey confocal (Thermo Noran, USA) fitted to a Nikon E600FN upright microscope. Averages of eight whole field images (206 µm × 158 µm) were routinely acquired every 5 s with a 40× objective lens (NA = 0.8). Acquisition and image analyses were performed using Noran Intervision and Metamorph software. Fluorescence values over time for specific regions of interest were exported into sigmaplot (Jandel, USA) for further analysis. The number of responding astrocytes (reported in the text and figures) is expressed as an absolute number of responding astrocytes per imaged slice area. Because the imaged area (see above) was the same in all experiments, and astrocytes are evenly distributed throughout the brain parenchyma [46], using the absolute number of responding astrocytes provides a valid and efficient way of determining agonist and antagonist effects as reported previously [47,48].

Two photon laser scanning microscopy was performed using a Prairie Ultima (Prairie Technologies, Madison, WI) microscope and a Ti : sapphire pulsed laser (Chameleon Ultra II, Coherent, UK) tuned to *λ* = 810 nm. Image acquisition was controlled using Prairieview software, and laser intensity was modified using a Pockels cell electroacoustic modulator (ConOptics, USA). Slices were imaged using a 40×/0.8 NA objective lens, and fluorescence signals from Fluo-4 indicator were collected in the epicollection mode using multi-alkali photomultiplier tubes (Hamamatsu Photonics, Hamamatsu, Japan).

### (c) Electrophysiology

Patch-clamp recordings from VTA neurons were made using pipettes (2–4 MΩ) containing an internal solution of composition (in mM): KMeSO_4_ 120, HEPES 10, EGTA 0.1, Na_2_ATP 4, GTP 0.5, osmolarity adjusted to 295 mOsm l^−1^ with KCl. Currents were recorded at −60 mV using a multi-clamp 700B amplifier, digitized with a Digidata 1440A, and acquired and analysed using pClamp (Molecular Devices). Neurons with more than 20% change in access resistance were excluded. Slow inward currents (SICs) were analysed using the event detection protocols in the Clampfit routine of pClamp. Events were accepted as SICs if their amplitude was more than 20 pA and their time to peak was more than 20 ms [47,49]. Data were exported to SigmaPlot (Jandel) for additional analysis and plotting.

### (d) Statistics

All quantitative data are expressed in the text as mean ± s.e.m. Statistical test used was unpaired Student's *t*-test. Dose–response curve fitting was conducted using the fitting procedures of SigmaPlot (Jandel) and Prizm (GraphPad). In the figures, asterisk indicates significance of *p < 0.05, **p < 0.005 and ***p < 0.0005.

## 3. Results

**Figure 1.**
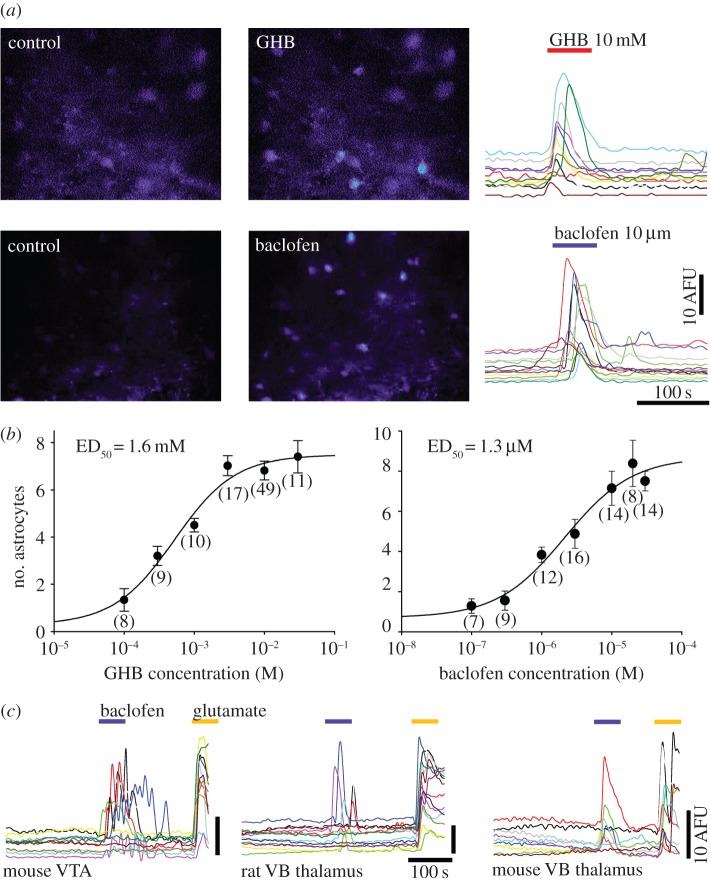
GHB and baclofen elicit [Ca^2+^]_i_ transients in VTA and thalamic astrocytes. (*a*) Fluorescence images of Fluo-4-loaded rat VTA slices before (control) and during a brief bath application of 10 mM GHB (top) or 10 µM baclofen (bottom) show robust and synchronous [Ca^2+^]_i_ transients in response to both drugs. The transients evoked in astrocytes in the two experiments are shown in fluorescence plots on the right. (*b*) Dose–response curves of the number of astrocytes responding to GHB and baclofen (number of slices is indicated below each data point). (*c*) Fluorescence plots from different preparations of mouse and rat VTA and VB thalamus show that GABA_B_R activation resulting in [Ca^2+^]_i_ elevations is a general mechanism, i.e. it can be observed in different species and brain regions. Glutamate (100 µM) applications are also shown for comparison. (Online version in colour.)

In the continuous presence of TTX (1 µM), relatively brief (2 min) application of GHB to rat VTA slices bulk-loaded with the Ca^2+^ indicator Fluo-4 elicited robust and synchronous somatic [Ca^2+^]_i_ transients in astrocytes (figure 1*a*, top row and the electronic supplementary material, movie S1), with increasing numbers of cells responding to increasing agonist concentration. The magnitude of GHB-induced elevations was not different in magnitude to those evoked by the selective GABA_B_R agonist baclofen (figure 1*a*, bottom row). These [Ca^2+^]_i_ elevations were present in the soma and in the fine astrocytic processes (electronic supplementary material, movie S1), and sometimes seen in processes without an apparent clear somatic response. Moreover, the GHB- and baclofen-elicited [Ca^2+^]_i_ transients could be observed in the mouse VTA (figure 1*c*) as well as in the rat nucleus accumbens (not shown) and in the mouse and rat VB thalamus (figure 1*c*). The astrocytic effect of GHB and baclofen was concentration dependent with an ED_50_ of 1.6 mM and 1.3 µM, respectively (figure 1*b*), with 10 mM GHB and 10 µM baclofen evoking [Ca^2+^]_i_ transients in 6.82 ± 0.39 astrocytes (*n* = 11 slices) and 7.14 ± 1.14 astrocytes (*n* = 14 slices), respectively.

**Figure 2.**
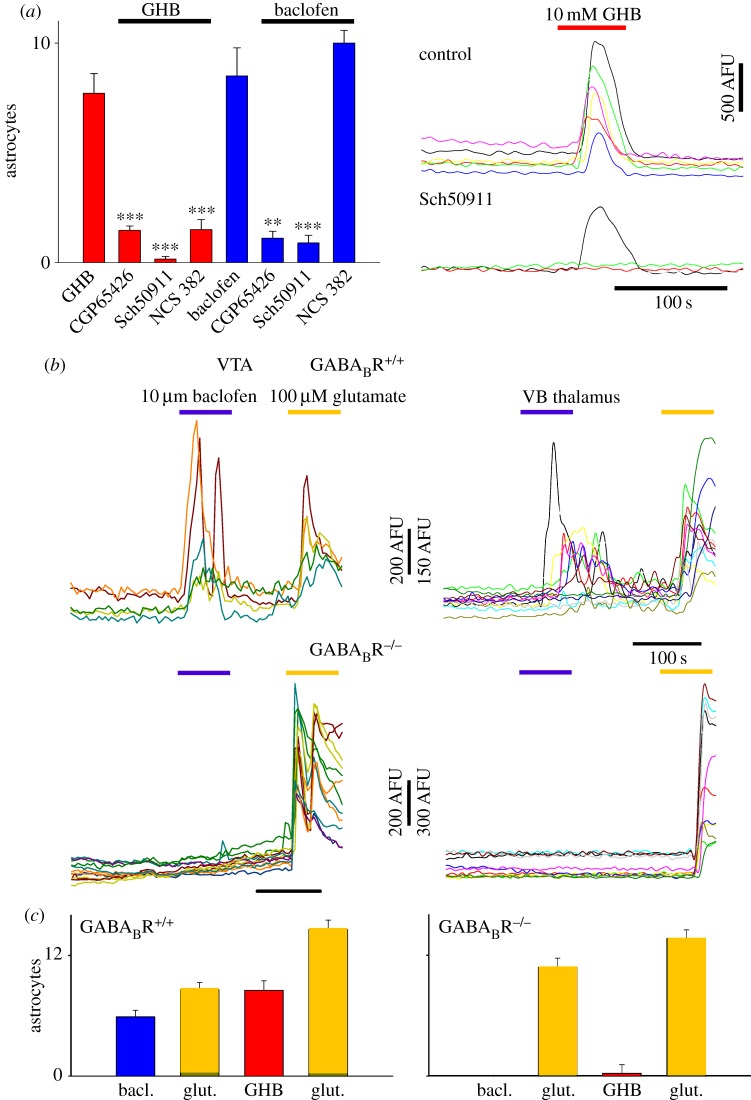
GHB and baclofen elicit astrocytic [Ca^2+^]_i_ elevations acting via GABA_B_Rs. (*a*) Summary bar graphs showing the number of astrocytes responding to a brief bath-application of 10 mM GHB and 10 µM baclofen alone or together with one of different GABA_B_R antagonists (as indicated). Astrocyte responses to both drugs are abolished by the two GABA_B_R antagonists, CGP65426 and Sch50911, whereas the GHB effect, but not that of baclofen, is also inhibited by the putative GHB receptor antagonist NCS382. Example fluorescence traces are shown on the right. (*b*) Fluorescence traces from experiments in mouse VTA slices (left column) show that astrocytes from WT littermates respond to baclofen, whereas in GABA_B_R KO mice baclofen responses are absent though astrocytes still respond to a 100 µM glutamate application. Similar results are seen in the VB thalamus (right column), indicating common mechanisms for astrocyte [Ca^2+^]_i_ elevations in different brain areas. (*c*) Summary bar graphs of similar experiments as in (*b*) showing number of responding astrocytes conducted in VTA for WT (left) and GABA_B_R KO (right) astrocytes with GHB and baclofen. (Online version in colour.)

To confirm that GABA_B_Rs were indeed mediating the astrocytic GHB and baclofen responses described above, experiments were conducted using pharmacological and transgenic interventions. Both GHB and baclofen effects were virtually abolished by two structurally different GABA_B_R antagonists, CGP5426 and SCH50911 (baclofen: *p* < 1 × 10^−5^, *p* < 1 × 10^−3^, respectively; GHB: *p* < 1 × 10^−7^, *p* < 1 × 10^−5^, respectively; figure 2*a*). Interestingly, GHB responses were also markedly inhibited by the putative GHB receptor antagonists NCS382 (GHB: 7.71 ± 0.89 astrocytes, *n* = 19 slices; GHB + NCS382: 1.5 ± 0.46, *n* = 8, *p* < 1 × 10^−4^; figure 2*a*), which, however, had no effect on the number of astrocytes responding to baclofen application (baclofen: 8.5 ± 1.28, *n* = 8 slices; baclofen + NCS382: 10 ± 0.57, *n* = 3 slices). Finally, astrocytes in VTA slices from wild-type littermate mice (WT, GABA_B_R^+/+^) readily responded with [Ca^2+^]_i_ elevations to GHB and baclofen applications, whereas both agonists failed to elicit astrocytic transients in slices from GABA_B_R knockout (GABA_B_R KO,^−/−^) mice (figure 2*b,c*). However, astrocytes in GABA_B_R KO and WT littermate mice responded similarly to glutamate (figure 2*b,c*), thus excluding the possibility that the mechanism underlying [Ca^2+^]_i_ transients in astrocytes from the GABA_B_R KO mice was compromised. Identical results were obtained in the VB thalamus of GABA_B_R KO and WT littermate mice (figure 2*b,c*).

**Figure 3.**
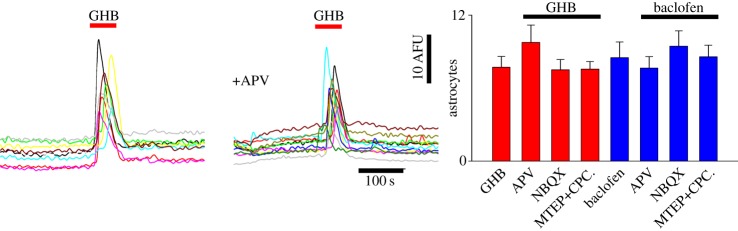
GHB- and baclofen-elicited astrocytic [Ca^2+^]_i_ transients are unaffected by block of glutamate receptors. Fluorescence traces show responses of astrocytes in a VTA slice to 10 mM GHB in control conditions and in the presence of APV. The summary bar graph on the right quantifies the lack of effect of NMDA, non-NMDA and mGluR antagonists (APV, NBQX and MTEP+ CPCCOEt, respectively) on the number of astrocytes responding to application of 10 mM GHB or 10 µM baclofen. (Online version in colour.)

Although all our experiments were conducted in the presence of TTX to block neuronal activity, the possibility might exist that the observed effect could be owing to activation of neuronal GABA_B_Rs that resulted in glutamate release and an indirect effect mediated by the activation of metabotropic glutamate receptors (mGluRs), which comprise a major signalling pathway in astrocytes [50]. As shown in figure 3, however, the response of VTA astrocytes to both GHB and baclofen was not affected by APV, NBQX or by the combined application of mGluR5 and mGluR1 antagonists (MTEP and CPCCOEt, respectively).

To investigate the sources of signalling elicited by GHB activation, we manipulated extracellular and intracellular [Ca^2+^]. To deplete intracellular stores, we used the SERCA pump inhibitor cyclopiazonic acid (CPA; 10 µM), whereas in separate experiments we tested the action of GHB and baclofen in slices bathed with a nominal extracellular Ca^2+^-free solution. Responses to baclofen were significantly reduced by both interventions (ctrl: 5.75 ± 0.70 astrocytes, *n* = 12 slices; CPA: 0.3 ± 0.15, *n* = 12, *p* < 1 × 10^−8^; 0 mM Ca^2+^: 0.22 ± 0.11, *n* = 12, *p* < 1 × 10^−8^) as were those to GHB (ctrl: 5.6 ± 0.56, *n* = 12; CPA: 0.28 ± 0.88, *n* = 16, *p* < 1 × 10^−5^; 0 mM Ca^2+^: 0.46 ± 0.13, *n* = 12, *p* < 1 × 10^−5^), showing that the effects of both agonists are dependent on Ca^2+^ release from intracellular stores and an extracellular Ca^2+^ supply, as previously described for similar [Ca^2+^]_i_ transients observed spontaneously and evoked by other neurotransmitter agonists [51,52].

**Figure 4.**
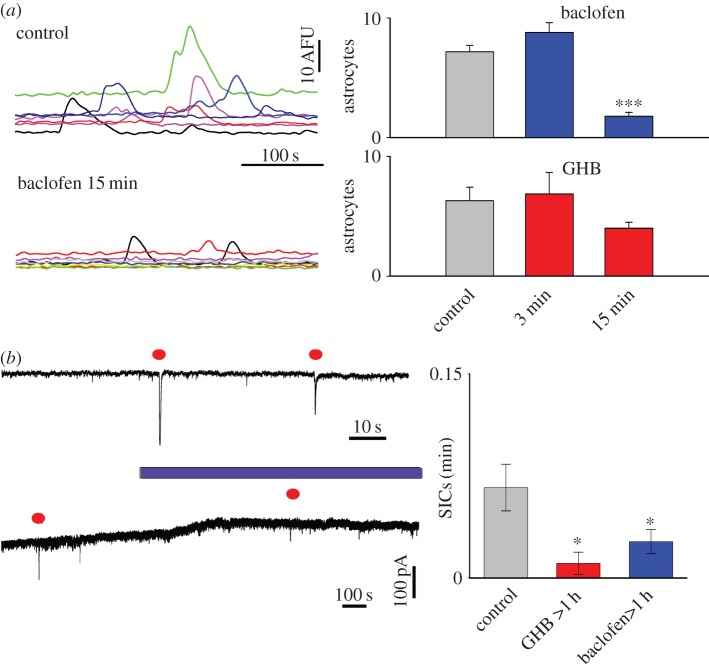
Time-dependent effect of GHB and baclofen on astrocytic [Ca^2+^]_i_ signalling and glutamate release. (*a*) Fluorescence traces show spontaneous astrocytic [Ca^2+^]_i_ transients in control conditions and in a VTA slice pre-exposed to 10 µM baclofen for 15 min. Bar graphs on the right summarize data from similar experiments where slices were pre-exposed to either 10 µM baclofen or 10 mM GHB for 3 and 15 min. (*b*) Patch-clamp recording from a VTA neuron on an expanded timebase illustrating spontaneous SICs in control conditions (top trace), and lower trace showing effect of long duration 10 µM baclofen application (SICs highlighted by filled circle). (*c*) Summary data showing the frequency of SICs in control conditions, and following long (>1 h) exposure to either 10 mM GHB or 10 µM baclofen. (Online version in colour.)

We have previously shown that the sustained stimulation of G-protein coupled mGluRs either by synaptic glutamate release or by agonist exposure results in an increase in the frequency of spontaneous astrocyte [Ca^2+^]_i_ oscillations which is associated with a [Ca^2+^]_i_-dependent increase in the frequency of astrocytic glutamate release events, i.e. neuronal SICs [49,53]. To investigate whether a similar phenomenon resulted from sustained GABA_B_R activation, we applied GHB and baclofen for increasing durations to VTA slices. Although our experiments show that a 2 min baclofen application elicited astrocyte [Ca^2+^]_i_ elevations (figures 1–3), a 3 min pre-exposure did not lead to an increase in the number of spontaneous astrocytic [Ca^2+^]_i_ transients, whereas following a 15 min pre-exposure to baclofen spontaneous astrocyte activity was reduced compared with control slices (ctrl: 7.16 ± 0.54 astrocytes, *n* = 8 slices; baclofen 15 min: 1.78 ± 0.32, *n* = 12, *p* < 1 × 10^−5^; figure 4*a*). Exposure to GHB also resulted in a reduction that approached statistical significance (ctrl: 6.3 ± 1.12, *n* = 12, GHB 15 min: 4.0 ± 0.5, *n* = 12, *p* = 0.069; figure 4*a*). To test whether these changes in spontaneous [Ca^2+^]_i_ signalling were translated to spontaneous astrocyte–neuron glutamatergic signalling recorded as SICs, we therefore treated VTA slices with baclofen or GHB for extended periods of 1–3 h and compared these results with those of untreated slices. Patch-clamp recordings made from VTA neurons in the presence of TTX showed the presence of spontaneous SICs in the VTA, confirming the existence of spontaneous astrocyte–neuron signalling in this brain area (figure 4*b*). Short (3 min) pre-incubation of the slice with baclofen application, however, did not induce astrocytic glutamate release as measured by SIC recording (ctrl: 0.056 ± 0.016 SICs per min, *n* = 11 slices; baclofen: 0.086 ± 0.027, *n* = 11, *p* = 0.2). Nevertheless, in agreement with the observed decrease in astrocytic [Ca^2+^]_i_ elevations observed during prolonged GABA_B_R activation (1–3 h), SIC frequency was significantly reduced by sustained pre-exposure to GHB and baclofen (ctrl: 0.066 ± 0.017 SICs per min, *n* = 28 slices; baclofen: 0.026 ± 0.009, *n* = 26, *p* < 0.05; GHB: 0.01 ± 0.008, *n* = 11, *p* < 0.05; figure 4*c*, right plot).

## 4. Discussion

The main findings of this study are that GHB consistently elicits robust [Ca^2+^]_i_ transients in astrocytes of both the VTA and the VB thalamus of young rodents, two brain areas that are involved in the reward properties and the pro-absence effect of this drug, respectively. This astrocytic GHB action is mediated by GABA_B_Rs and is comparable to that evoked in astrocytes of the same regions by the selective GABA_B_R agonist baclofen. Moreover, prolonged GHB and baclofen exposure causes a reduction in spontaneous glutamate release from VTA astrocytes.

### (a) Astrocytic gamma-hydroxybutyric acid effects

The astrocytic response to GHB (and baclofen) was highly reliable and consistent from trial to trial in the age range tested, and thus did not show the variability that has been reported in a previous study [42]. Determination of whether there is a difference in age-dependent GABA_B_R signalling that is also region-dependent will require further studies in the VTA and VB thalamus.

Both GHB- and baclofen-elicited [Ca^2+^]_i_ transients were (i) abolished by two highly selective but structurally dissimilar GABA_B_R antagonists, (ii) absent in GABA_B_R KO mice and (iii) unaffected by the block of ionotropic and metabotropic glutamate receptors, indicating that in astrocytes, as occurs in neurons, the action of GHB is mediated by GABA_B_Rs. Indeed, the 1000-fold difference in the ED_50_ of the two drugs on astrocytic responses is very similar to that reported for their activation of pre- and post-synaptic neuronal GABA_B_Rs, including hyperpolarization of the membrane potential and inhibition of synaptic potentials [54]. However, the astrocytic effect of GHB was also markedly blocked by NCS382, an antagonist of the putative GHB receptor. This represents the first solid evidence of a CNS action of GHB being mediated by its putative receptor [3], and might provide an explanation for the block of GHB-elicited absence seizures observed in some studies [34]. Finally, the lack of action of ionotropic and metabotropic glutamate receptors on GHB-elicited [Ca^2+^]_i_ transients indicates that these responses are not mediated by activation of astrocytes by glutamate, secondary to neuronal activation, and suggests that they results from direct activation of astrocytic GABA_B_Rs.

GHB- (and baclofen)-elicited [Ca^2+^]_i_ transients were blocked following perfusion of VTA slices with either a nominal extracellular Ca^2+^-free solution or with CPA. Thus, our results indicate that both GHB and baclofen astrocytic responses in VTA involve intracellular Ca^2+^ stores and their refilling by extracellular Ca^2+^. Whether GHB activity requires G_i/o_ proteins (which are classically associated with neuronal GABA_B_R activation) or G_q_ proteins (which have been strongly linked to Ca^2+^ release from intracellular stores) remains to be elucidated, as is indeed the case for the astrocytic response to baclofen in VTA or any other brain region.

Finally, in contrast to the activation of astrocytes, brief application of GHB and baclofen had no effect on spontaneous [Ca^2+^]_i_ transients (see control grey bar in figure 4*a*), but decreased their frequency when applied for periods of 15 min or longer. A similar picture was obtained when looking at spontaneous SICs, the neuronal counterpart of astrocytic released glutamate, suggesting that long-term activation of GABA_B_R lead to a reduction in the glutamate-dependent astrocyte to neuron signalling.

### (b) Potential (patho)physiological implications of gamma-hydroxybutyric acid activation of astrocytes

Our finding of an astrocytic activation by GHB opens new avenues in the interpretation of the effects of this substance. As far as absence seizures are concerned, our results suggest for the first time that the ability of GHB to induce these seizures might involve an astrocytic component together with a neuronal component. This, together with the already described loss of function of GAT1 in thalamic astrocytes of genetic models of absence seizures [29] and the fact that GABA transporters affect astrocytic [Ca^2+^]_i_ transients [55], clearly stresses the importance of this glial cell type in the generation of these non-convulsive seizures. Finally, our data showing a block of GHB-elicited astrocytic [Ca^2+^]_i_ transients by NCS382 provide a potential explanation for the anti-absence effect of this putative GHB receptor antagonist, the action of which had so far been difficult to reconcile with its lack of action on neuronal response in thalamus [3], one of the key regions responsible for the generation of these non-convulsive seizures.

Drugs that cause addiction act in the VTA to increase dopamine release in target areas, notably the nucleus accumbens. It has been suggested that GHB achieves this by inhibiting GABAergic interneuron activity which results in disinhibition of dopaminergic neurons in the VTA, and so increased dopamine release [56,57]. Our observations suggest a possible astrocytic contribution to such a mechanism, if a reduced astrocytic glutamate release which may ordinarily contribute to tonic glutamate has a physiological role in driving GABAergic interneuron activity. Reducing such a drive would therefore be expected to increase the output of dopaminergic neurons. There are reports from many brain areas of a Ca^2+^-dependent gliotransmitter release increase following the activation of G-protein coupled receptors [58]: these examples are linked to PLC and IP_3_ production via G_q_, whereas GABA_B_Rs are coupled to adenylyl cyclase via G_i/o_ [54]. We found that in astrocytes, GABA_B_R activation elicits [Ca^2+^]_i_ elevations. The mechanism underlying this increase is unclear but may involve some activation of G_q_-coupled pathways, or novel interactions between GABA_B_ auxiliary subunits and intracellular Ca^2+^ release pathways. Whatever the physiological role of the GABA_B_R-induced [Ca^2+^]_i_ elevations, it does not seem to invoke glutamate gliotransmitter release. It does, however, provide an experimental reporter of astrocyte GABA_B_R activation, and our observations are consistent with dominant activation of non-G_q_ pathways, because we found a reduction in spontaneous gliotransmitter glutamate release (i.e. SICs). Interestingly, this observation is analogous to the effect of GABA_B_R activation on inhibiting spontaneous presynaptic neurotransmitter release [59], and indicates the complexity of signalling pathways which may influence the ways that astrocytes interact with neuronal activity in different brain areas.
